# Rickettsial Infections and Q Fever Amongst Febrile Patients in Bhutan

**DOI:** 10.3390/tropicalmed3010012

**Published:** 2018-01-25

**Authors:** Tshokey Tshokey, John Stenos, David N. Durrheim, Keith Eastwood, Chelsea Nguyen, Gemma Vincent, Stephen R. Graves

**Affiliations:** 1 Faculty of Health and Medicine, University of Newcastle, Callaghan, NSW 2308, Australia; david.durrheim@newcastle.edu.au (D.N.D.); keith.eastwood@hnehealth.nsw.gov.au (K.E.); graves.rickettsia@gmail.com (S.R.G.); 2 Australian Rickettsial Reference Laboratory (ARRL), University Hospital Geelong, Geelong 3220, Australia; johns@barwonhealth.org.au (J.S.); chelsean@barwonhealth.org.au (C.N.); gvince@barwonhealth.org.au (G.V.); 3 Jigme Dorji Wangchuck National Referral Hospital (JDWNRH), Thimphu 11001, Bhutan; 4 Population Health, Hunter New England Local Health District, New Lambton, NSW 2305, Australia

**Keywords:** Bhutan, Q fever, rickettsial infections, scrub typhus, undifferentiated fever

## Abstract

There is limited evidence of rickettsial diseases in Bhutan. We explored the contribution of rickettsioses as a cause of undifferentiated febrile illness in patients presenting to 14 Bhutanese hospitals from October 2014 to June 2015. Obvious causes of fever were excluded clinically. Clinico-demographic information and acute blood samples were collected. Samples were tested by immunofluorescence assay (IFA) and qPCR against scrub typhus group (STG), spotted fever group (SFG) and typhus group (TG) rickettsiae, and Q fever (QF). Of the 1044 patients, 539 (51.6%) were female and the mean age was 31.5 years. At least 159 (15.2%) of the patients had evidence of a concurrent rickettsial infection. Of these, 70 (6.7%), 46 (4.4%), 4 (0.4%), and 29 (2.8%) were diagnosed as acute infections with STG, SFG, TG, and QF respectively. Ten (1.0%) patients were seropositive for both SFG and TG. Seven of the 70 STG patients were positive by qPCR. Eschar (*p* < 0.001), myalgia (*p* = 0.003), and lymphadenopathy (*p* = 0.049) were significantly associated with STG, but no specific symptoms were associated with the other infections. Disease incidences were not different between age groups, genders, occupations, and districts, except for students with significantly lower odds of infection with STG (OR = 0.43; 95% CI = 0.20, 0.93; *p* = 0.031). Rickettsioses were responsible for at least 15% of undifferentiated febrile illnesses in Bhutan, scrub typhus being the commonest. Health authorities should ensure that health services are equipped to manage these infections.

## 1. Introduction

*Rickettsia*, *Orientia,* and *Coxiella* cause undifferentiated febrile illnesses of varying severity in humans. Diseases caused by *Rickettsia* and *Orientia* species are together referred to as rickettsioses, and Q fever (QF) caused by *Coxiella burnetii* is frequently included under rickettsioses [1]. Rickettsial diseases are both emerging [2] and re-emerging [3]. The genus *Rickettsia*, with more than 22 species, comprises several groups, two of which, the spotted fever group (SFG) and typhus group (TG), are the most important causes of human disease [4]. *Orientia*, with two known species, *O. tsutsugamushi* and *O. chuto* [5], form the scrub typhus group (STG). SFG and TG have a worldwide distribution and STG is mainly found in the Asia-Pacific region [1]. Human infection with QF occurs worldwide [6] except in New Zealand [7]. 

The detection and diagnosis of rickettsial diseases remains inadequate and is not widely available. Serological techniques can provide difficulties in interpretation and may cross-react with other antigens. However, serology is the most utilized technique and immunofluorescence assay (IFA) is regarded as the current gold standard. For diagnosis, the presence of IgM antibody indicates an acute/primary infection and IgG appears about two weeks after the infection. IgM may be present for months and IgG may remain detectable for years [8,9], thus confusing the diagnosis of an acute infection. The examination of both acute and convalescent sera is usually required for confirming an acute diagnosis [9]. Molecular techniques have been used for increasing the sensitivity/specificity of diagnosis, in identifying new rickettsial species, and also in epidemiological studies [8]. Rickettsial diseases are believed to be a significant cause of morbidity in south-east Asia [10]. Scrub typhus has been reported widely from countries neighboring Bhutan, with recent increases in cases and outbreaks [3,11,12,13]. There is limited recent data on Q fever in humans in the region compared to other rickettsial diseases. In Bhutan, scrub typhus is better known than other rickettsial infections. There were two recorded outbreaks of scrub typhus; one in 2009 [14] prompting preliminary scrub typhus surveillance [15], and another in 2014 [16,17]. A review noted that scrub typhus is being increasingly reported in Bhutan, with 91 cases in 2010 and 351 cases in 2013 [16], increasing to 605 cases in 2015 [18]. Although awareness is improving, there are no clinical guidelines supporting the diagnosis and treatment of rickettsial diseases in Bhutan. The first published seroprevalence study for Bhutan reported an overall seroprevalence of 49% against rickettsioses; the commonest being STG (22.6%), followed by SFG (15.7%), Q fever (6.9%), and TG (3.5%) [19]. There is still a huge gap in the understanding of causes of febrile illnesses in Bhutan. This current study was undertaken to investigate the potential contribution of rickettsial diseases amongst patients presenting with undifferentiated fever attending hospitals in Bhutan. 

## 2. Materials and Methods

### 2.1. Setting and Study Sites

**Figure 1:**
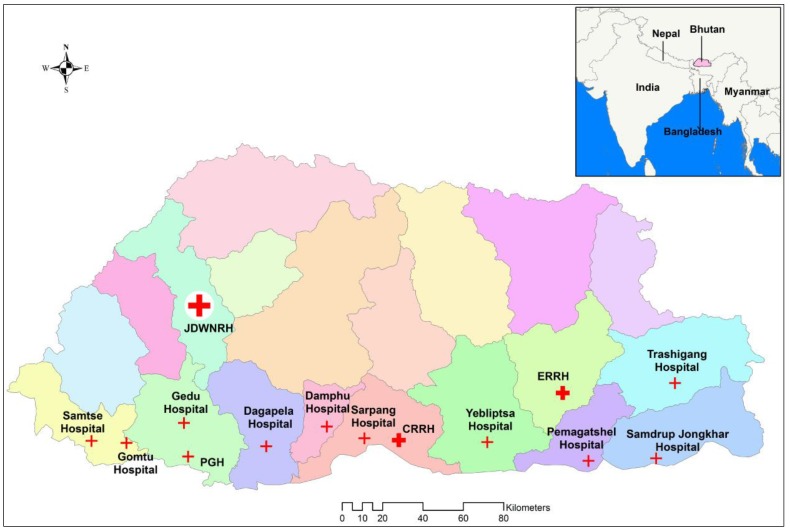
Map of Bhutan showing the 14 hospitals (study sites).

Bhutan is comprised of 20 administrative districts with an estimated population of 770,000 in 2016 [20]. Due to the landscape and harsh terrain, district population density varies between 9 and 64 people/km^2^ [21]. The country has over 70% forest cover and about 70% of the population lives in rural areas. With four distinct seasons, the mean daily temperature varies between 5 °C in the winter to 25 °C in the summer. The annual average rainfall varies from less than 500 mm in the northern Himalayas, to 500–1000 mm in the inner central valleys, and 2000–5000 mm in the southern foothills [22]. Each district has one or more hospitals and medical treatment is provided free of charge by the government. Fourteen of the 28 hospitals in the country (Figure 1) were selected for this study. The hospitals were intentionally selected based on their size (larger hospitals), location (ensuring the more populous south-central region was appropriately represented), and previous records of scrub typhus occurrence. The 14 hospitals were located in 11 of the 20 districts, but with a combined estimated population of 573,000, they provide health services to about 75% of Bhutan’s population [20]. 

### 2.2. Study Design and Participants

A prospective descriptive study was carried out from October 2014 to June 2015. Patients (inpatients and outpatients) attending the 14 hospitals with ‘undifferentiated fever of ≥4 days duration accompanied by one or more symptoms of headache, chills, myalgia, arthralgia, lymphadenopathy, or skin rash/eschar’ were included in the study after excluding other obvious systemic or local causes of fever (such as respiratory tract infections, urinary tract infections, abscesses and cellulitis, otitis media, etc.) through clinical examination. Clinico-demographic information was recorded and a single acute EDTA blood sample and a serum sample was collected from each patient. No convalescent sera and treatment details were collected and patients were not followed up for treatment outcomes. Blood samples were stored at −70 °C until their shipment to the Australian Rickettsial Reference Laboratory (ARRL), an accredited reference center for rickettsial testing [23].

### 2.3. Laboratory Testing

At the ARRL, all samples were tested for antibodies to STG, SFG, TG, and QF by IFA [24], and for rickettsial (STG, SFG, and TG) and *Coxiella* DNA by qPCR. Antibodies against STG were tested using *O. tsutsugamushi* (Gilliam, Karp, and Kato strains) and *O. chuto* antigens; against SFG using *R. australis*, *R. honei*, *R. conorii*, *R. africae*, *R. rickettsii,* and *R. felis* antigens; against TG using *R. prowazekii* and *R. typhi* antigens; and against QF using *C. burnetii* phase I and II antigens. All antigens were prepared in-house in the ARRL by culturing the respective organisms in L929 cell line and RPMI media (Invitrogen) with 5% fetal bovine serum (FBS). Samples were screened at low dilutions and titrated to end titre if positive. Positive and negative control antigens were included and tested with every slide. 

DNA was extracted from the buffy coat sample using a HiYield^TM^ DNA Mini Kit, YGB100, Real Genomics (Taipei, Taiwan), and tested using qPCR procedures established as the ARRL protocol [25,26]. Any samples with Ct values of <35 were deemed positive, between 35 and 40 equivocal (repeated to determine their status), and >40 negative, against the specific rickettsial agents. SFG and TG being genetically similar, were tested targeting the citrate synthase (CS) gene (CS-F 5′-TCG CAA ATG TTC ACG GTA CTT T-3′, CS-R 5′-TCG TGC ATT TCT TTC CAT TGT G-3′, CS-Probe 5′-FAM TGC AAT AGC AAG AAC CGT AGG CTG GAT G BHQ1-3′) [26]. The *16S rDNA* gene was targeted for *Orientia* [27] (16S rDNA-F 5′-CTT ATT TGC CAG CGG GTA ATG C-3′, 16S rDNA-R 5′-GGG CCA TGA TGA CTT GAC CTC-3′, 16S rDNA-Probe 5′-FAM CCC ACC TTC CTC CGG CTT AGC ACC BHQ1-3′) and the com 1 gene for *Coxiella* [27] (com1-F 5′-AAA ACC TCC GCG TTG TCT TCA-3′, com1-R 5′-GCT AAT GAT ACT TTG GCA GCG TAT TG-3′, com1-probe 5′-FAM AGA ACT GCC CAT TTT TGG CGG CCA BHQ1-3′), both designed using Primer Express (Applied Biosystems, Foster City, CA, USA). Attempts at sequencing the DNA from the qPCR positive samples were not successful.

### 2.4. Defining Current Infections

In primary rickettsial infections, antibody responses usually appear by the second week of illness. While acute infections are best diagnosed by evidence of rising antibody titres (ideally by a four-fold rise) between acute and convalescent samples, it was not possible in this study with no convalescent sera available. When ascertaining acute infections using single acute serum samples, interpretation is potentially confounded by interfering background antibodies from previous exposures, especially in endemic areas [28], and high background seroprevalence has been proven to exist in Bhutan [19]. For diagnosing a current infection in this study, a high titre of IgM antibody with adequate time (≥14 days of fever) for antibody development was considered appropriate. IgM antibody titres of ≥1:1024 were considered positive for *Rickettsia* (SFG and TG) and *Orientia* (STG), while IgM titres of ≥1:100 against *C. burnetii* phase I or II antigens or both were considered positive for Q fever. These titres were chosen at least four-fold above the standard positive titres of these assays to ensure confidence of the causative infection. When there was sero-reactivity against two or more antigen groups, the higher titre was considered the likely current infection. When the qPCR was positive, infection was considered current regardless of duration of illness and antibody titre since the presence of DNA denotes current infection.

### 2.5. Statistical Analysis and Determination of Associations

Data were analyzed using STATA software version 14. Clinical and demographic variables of interest were reported descriptively in frequencies and percentages. Associations between infections and patients’ age groups, occupations, districts, and environmental exposures were explored using the Chi-squared test or Fisher’s test as appropriate, considering a *p* value of ≤0.05 significant. Univariate logistic regression analysis was used to determine odds ratios (OR), and *p* values of <0.05 were considered significant for association of the variables against the infections. 

### 2.6. Ethics, Consent and Confidentiality

This study was approved by the Research Ethics Board of Health (REBH), Bhutan (Ref: REBH/Approval/2014/019), and the Human Research Ethics Committee (HREC), University of Newcastle, Australia (Ref: H-2016-0085). All patients or parents/guardians agreed to participate in the study and provided informed consent before participation. All information and samples were anonymized.

## 3. Results

### 3.1. Demography

From a total of 1044 patients, 539 (51.6%) were females. With a mean age of 31.5 years (95% CI, 30.4, 32.5) and median age of 30 years, the youngest patient was one year of age and the oldest 88 years old. There was an almost equal number of farmers, office workers, and students (Table 1). 

The 14 hospitals enrolled a median of 74.5 cases, with the highest enrolment of 122 patients from the Jigme Dorji Wangchuck National Referral Hospital (JDWNRH), Thimphu district, and the lowest of 46 from Damphu hospital, Tsirang district. Patients enrolled in the study had an overall mean of 6.8 days of fever (95% CI, 6.0, 6.5), with a maximum period of illness of 30 days. 

### 3.2. Laboratory Findings

**Figure 2:**
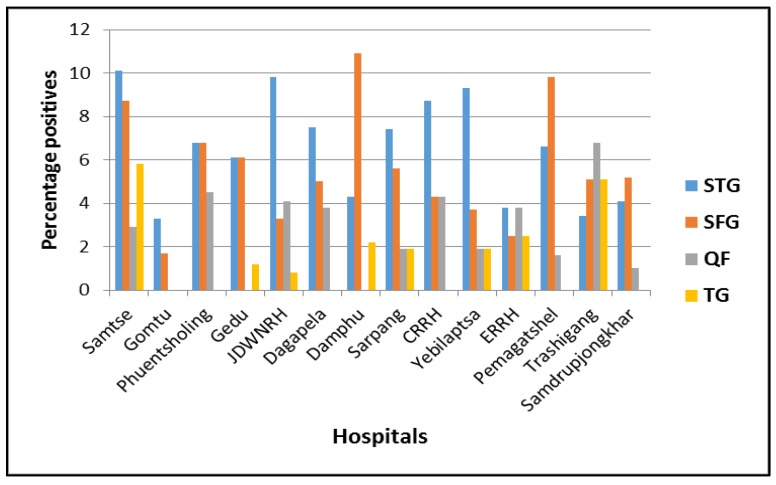
Overall seropositivity of the four infections in the 14 hospitals (STG: scrub typhus group; SFG: spotted fever group; QF: Q fever; TG: typhus group).

Of the 1044 patients, 70 (6.7%), 46 (4.4%), 4 (0.4%), and 29 (2.8%) were positive against STG, SFG, TG, and QF respectively. Ten (1.0%) patients had equal antibody titres against SFG and TG. Seven of the 70 patients positive for STG were tested positive by qPCR (4 positive only by qPCR and 3 positive by both qPCR and IFA). All other samples were tested negative by qPCR for SFG and TG *Rickettsia*, *Orientia,* and *Coxiella* DNA. This resulted in an overall case incidence of 15.2% in the 14 hospitals during the study period. The distribution of cases by age and occupation is presented in Table 2, and individual hospital cases are presented in Figure 2. 

### 3.3. Clinical Presentations

The study recorded nineteen different presenting symptoms, including fever (100%, *n* = 1044), an essential inclusion criteria, headache (77%, *n* = 805), arthralgia (60%, *n* = 623), myalgia (29%, *n* = 298), rash (25%, *n* = 262), lymphadenitis (3%, *n* = 33), and eschar (3.8%, *n* = 40). Some patients also reported other symptoms like cough, anorexia, and backache. An eschar was seen in 44.3% (31/70) of STG patients, but not in acute cases of SFG, TG, and QF. The presence of an eschar (*p* < 0.001), myalgia (*p* = 0.003), and lymphadenopathy (*p* = 0.049) were significantly associated with STG (Table 3). No symptoms were statistically associated with the other infections. 

To compare signs and symptoms of rickettsioses to other non-rickettsial causes of undifferentiated fever, cases of all four infections (STG, SFG, TG, and QF) were pooled together (as rickettsioses) and their signs and symptoms compared with those negative patients (non-rickettsioses) as in Table 4. In this comparison, the presence of an eschar remained the only significant sign of a rickettsial infection (*p* < 0.001).

### 3.4. Environmental Factors and Association with Rickettsial Infection

Information on exposure to environmental factors such as domestic animals, pets, bush/forest, and tick/flea/mite bites was collected. About 47% (493/1044) reported bush/forest contact, 45% (472/1044) animal contact, 13% (118/1044) tick bites, and 11% (107/1044) flea/louse bites, Of those with animal contact, almost 60% (282/472) did not specify the animal species and cattle 35% (167/472) was the commonest animal of contact. None of these factors was significantly associated with any of the four infections.

In logistic regression analysis for the association of age, gender, occupation, district of residence, and other environmental risk factors, none was significant against SFG, TG, and QF infections. However, with STG there was significantly lower odds of infection in students (OR = 0.43; 95% CI = 0.20, 0.93; *p* = 0.031) compared to other occupational groups, as shown in Table 5. All other factors were insignificant.

**Figure 3:**
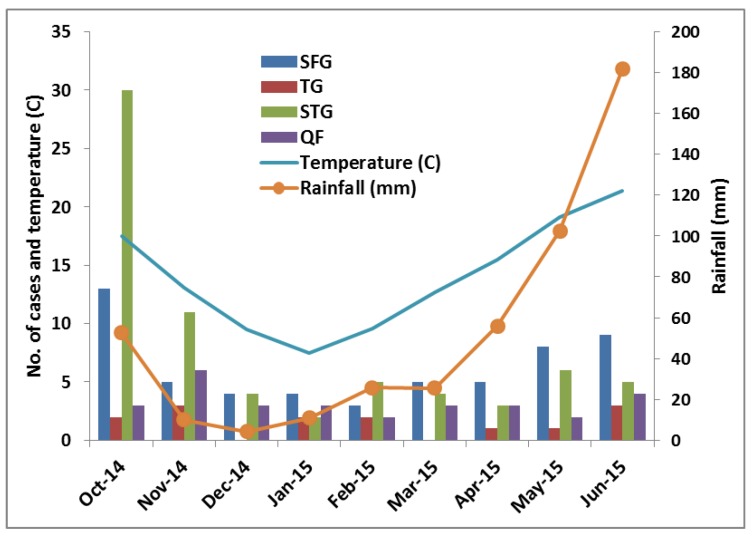
Cases by month in relation to average monthly temperature and precipitation.

The number of cases, particularly of STG and SFG, gradually decreased from October (30 STG and 13 SFG cases) to February (5 STG and 3 SFG cases), and then increased from March (5 STG and 4 SFG cases) to June (9 STG and 5 SFG cases), during and after the warm, rainy summer season. This occurrence correlated with the average monthly precipitation and temperature of Bhutan [29], indicating that STG and SFG occur mostly during the warm monsoon and following months (Figure 3 and Supplementary Table S1).

## 4. Discussion

Rickettsioses appeared to be an important infectious disease in the 14 Bhutanese hospitals during the nine-month study period (October 2014–June 2015). In about 15% (at least one in every six) of the patients presenting with an undifferentiated febrile illness, there was evidence of a concurrent rickettsial infection, scrub typhus being the commonest. Although inferences may have been tempered by testing only single acute serum samples, the use of a high IgM titre (≥1:1024) and only including patients with an adequate duration for antibody response after the onset of illness (≥14 days) likely provided a good estimate of current rickettsial infections in this study. Although scrub typhus has been increasingly recognized over the past decade with two reported outbreaks [16,17], this is the first study on SFG, TG, and Q fever in Bhutan amongst acute febrile patients in the hospital setting.

The mean duration of illness before seeking medical attention was 6.8 days and there were patients who visited hospitals as late as 30 days after the onset of illness (range 4–30 days). Neglect of treatment could have serious outcomes [14,16,17] in an otherwise easily treatable rickettsial infection. Such practices have huge implications in a highly spiritual and religious society like Bhutan, where some sections of society are still known to seek medical attention only after exhausting home remedies. Presenting signs and symptoms in this study were similar to studies from India [30,31], Taiwan [32], and Thailand [33]; fever (100%) being the most common followed by headache and arthralgia. Our study, however, did not record other signs and symptoms, such as jaundice, ocular congestion, hepatomegaly, and splenomegaly, that were reported as significant in other studies [34,35].

In Bhutan, a largely rural country with high livestock ownership, participants indicating bush/forest contact of only 47% and animal contact of only 45% (472/1044) was unexpected, and possibly underreported. Finding no association between environmental factors and these vector-borne diseases probably indicates that people who work in offices or attend schools may nevertheless come into regular contact with domestic animals and vegetation, albeit to a lesser extent than that of farmers. Students revealing lower odds of infection with STG in this study should be interpreted with caution. The degree to which students may be exposed to the infections is completely dependent on the location of schools, and environmental and living conditions can vary widely. Outbreaks of scrub typhus [17] have occurred in schools in remote parts of Bhutan in the past.

STG was the most commonly identified rickettsiosis in this study, similar to reports from Sri Lanka [36] and south India [37]. An eschar was seen in 44.3% (31/70) of STG patients in this study, compared to 93% (243/261) in a study in Taiwan [38], 40% (59/146) in Thailand [33], and 10% (2/21) in an Indian study [39]. Although current findings are similar to others, it is crucial to understand that eschars occur at the site of chigger bites in often concealed body areas and can be easily missed without a detailed physical examination. A Thai study reported eschars to be mostly located in the perineal, inguinal, and buttock areas in males, and on the head and neck in females [33]. The significant association of an eschar with STG positivity (*p* < 0.001) in this study substantiates its usefulness in making a clinical diagnosis of scrub typhus, if present. Additionally, myalgia (*p* = 0.003) and lymphadenopathy (*p* = 0.049) could be clinically useful diagnostic symptoms of scrub typhus in the Bhutanese setting. However, only eschar (*p* < 0.001), but not myalgia (*p* = 0.149) and lymphadenopathy (*p* = 0.145), would useful in differentiating rickettsioses from other non-rickettsial causes of undifferentiated febrile illnesses. 

SFG rickettsiosis was the second commonest infection in this study. This was in contrast to findings in central India [40] and Africa [41,42] where SFG cases were more common than STG amongst febrile patients. Because of fewer positive SFG cases, no significant correlations could be derived. In this study, *R. typhi* (causing endemic/murine typhus) and *R. prowazekii* (causing epidemic typhus) were individually tested but considered together as TG rickettsiae due to cross-reactivity in IFA. However, due to past evidence in Bhutan [15] and the non-epidemic nature of the current illnesses, the cases in this study were probably murine typhus. Epidemic typhus, once dreaded for causing epidemics, now seem to have become less prevalent than other rickettsial diseases as shown in this and other similar studies [40,41,42,43]. This could perhaps be due to a general improvement in living conditions and hygiene, resulting in reduced flea and louse infestations. However, certain populations, such as those in prisons, refugee camps, orphanages, and boarding schools, may still be at risk. Nevertheless, a study in Nepal’s capital, Kathmandu, detected 17% seropositivity against murine typhus [44]. The incidence of QF at 2.8% of acute infections in this study was lower than expected but reflects a study in Nepal, a country with similar geographical conditions to Bhutan, where only one of 125 acute undifferentiated fever patients tested positive (0.8%) for QF [44]. This low incidence could be explained by the fact that, although livestock ownerships is high, domestic animals (the common sources of infection) are primarily free-range and found only in small herds mostly restricted to small land holdings, unlike large high-density commercial farms in developed countries. Studies on coinfections of rickettsial diseases are limited, but dual infection with STG and QF was seen in 3.6% (5/137) of patients in Taiwan [32]. Although coinfections with these arthropod-borne diseases are possible, in our study, only 10 (1.0%) patients had equal antibody titres against SFG and TG rickettsiae. These were more likely due to cross-reacting antibodies rather than concurrent infections. 

As reported in other studies [16], STG and SFG rickettsioses showed seasonal variation with greater activity in wet, warmer months, but the current study was limited to nine months (October–June) due to time and financial constraints. However, seasonal variations were shown in previous studies in temperate regions [1,4]. The seasonality of SFG rickettsioses may be explained by the activity of the tick vectors, particularly the adults, which are more active during the spring and early summer [4], and that of STG due to the growth of vegetation following the warm monsoon [45]. 

In situations where diagnostic facilities are limited, greater clinical awareness and a higher index of suspicion among healthcare workers could increase case detection and reduce morbidity and mortality, as rickettsial infections respond rapidly to appropriate antibiotic therapy. A previous Bhutanese study recounted that many non-malarial, non-typhoidal febrile cases which responded readily to doxycycline or chloramphenicol may have been rickettsial infections [16]. For Bhutan, the increasing number of cases may be due to uncovering a longstanding neglected endemic situation rather than a re-emergence of the infections. Public health authorities should ensure that health services are equipped to diagnose and treat these cases. Further studies are indicated to explore risk factors and biosocial characteristics of disease transmission to identify appropriate preventive and control measures. Additionally, studies should attempt speciation of the rickettsial agents and the emergence of antibiotic resistance as detected in other countries. Similar studies of longer duration across all seasons with patient follow-up to obtain convalescent samples and response to treatment should confirm the results of the current study. 

This study has several important limitations. The case definition was overtly clinical and other possible causes of undifferentiated febrile illness such as malaria, typhoid fever, dengue fever, leptospirosis, and brucellosis were not excluded as test kits were not available. Additionally, only single acute blood samples were collected and tested, making it difficult for a definitive serological diagnosis of acute/current infection to be made due to background antibodies that are known to interfere with the diagnosis of the current infection. Because of the low positivity rate, especially of QF and TG rickettsioses, data should be interpreted with caution. 

## 5. Conclusions

This study, the first of its kind in Bhutan, found that at least 15% (one in every six) of the undifferentiated febrile illnesses in the 14 Bhutanese hospitals were due to a rickettsiosis, scrub typhus being the commonest. Since the 14 hospitals in the study represented about 75% of Bhutan’s population, the findings may likely be representative of the country, especially in the absence of any other similar studies at present. The findings of this study may warrant the Bhutan Ministry of Health to recognize the burden of rickettsioses and intervene to reduce their threat through the development of clinical guidelines and community education. 

## Figures and Tables

**Table 1:** Patient distribution by age group and occupation (*n* = 1044).

Occupation	Age Groups in Years					
	<13	13–24	25–36	37–48	>48	Overall (%)
Farmer	0	20	55	71	128	274 (26)
Office worker	0	22	134	82	31	269 (26)
Student	114	165	7	0	0	286 (27)
Housewife	0	17	62	33	37	149 (14)
Unemployed	0	5	5	4	2	16 (2)
Pre-school	50	0	0	0	0	50 (5)
Total (%)	164 (16)	229 (22)	263 (25)	190 (18)	198 (19)	1044 (100)

**Table 2:** Number of positive cases of each infection by age group and occupation.

Variable	No. of Positives (%)				
Age Group (yrs.)	STG (*n* = 70)	SFG (*n* = 46)	TG (*n* = 4)	SFG + TG (*n* = 10)	QF (*n* = 29)
<13	10 (14.2)	7 (15.2)	0 (0.0)	1 (10.0)	0 (0.0)
13–24	14 (20.0)	10 (21.7)	1 (25.0)	4 (40.0)	7 (24.1)
25–36	13 (18.6)	16 (34.8)	0 (0.0)	3 (30.0)	7 (24.1)
37–48	20 (28.6)	8 (17.4)	1 (25.0)	0 (0.0)	6 (20.7)
>48	13 (18.6)	5 (10.9)	2 (50.0)	2 (20.0)	9 (31.1)
Occupation					
Farmer	23 (32.9)	11 (23.9)	1 (25.0)	6 (60.0)	11 (37.9)
Office worker	21 (30.0)	9 (19.6)	2 (50.0)	1 (10.0)	8 (27.6)
Student	10 (14.3)	14 (30.4)	1 (25.0)	1 (10.0)	5 (17.2)
Housewife	10 (14.3)	8 (17.4)	0 (0.0)	1 (10.0)	3 (10.4)
Unemployed	1 (1.4)	3 (6.5)	0 (0.0)	0 (0.0)	2 (6.9)
Pre-school	5 (7.1)	1 (2.2)	0 (0.0)	1 (10.0)	0 (0.0)

**Table 3:** Association of signs and symptoms with STG, SFG, TG, and QF cases.

Signs and Symptoms		STG (*n* = 70)			SFG (*n* = 56)			TG (*n* = 14)			QF (*n* = 29)		
		Pos	Neg	*p* Value	Pos	Neg	*p* Value	Pos	Neg	*p* Value	Pos	Neg	*p* Value
Rash	Yes	23	240	0.126	11	252	0.325	4	259	0.769	8	255	0.763
	No	47	734		45	736		10	771		21	760	
Eschar	Yes	31	9	<0.001 *	0	40	0.125	0	40	0.452	0	40	0.276
	No	39	965		56	948		14	990		29	975	
Headache	Yes	55	750	0.763	48	757	0.115	13	792	0.158	22	783	0.871
	No	15	224		8	231		1	238		7	232	
Arthralgia	Yes	48	575	0.116	37	586	0.316	11	612	0.147	19	604	0.515
	No	22	399		19	402		3	418		10	411	
Myalgia	Yes	31	267	0.003 *	17	281	0.761	3	295	0.551	5	293	0.171
	No	39	706		39	706		11	734		24	721	
Lymphadenopathy	Yes	5	28	0.049 *	2	31	0.859	0	33	0.496	1	32	0.930
	No	65	944		54	955		14	995		28	981	
Others	Yes	8	99	0.736	10	97	0.054	1	106	0.700	1	106	0.221
	No	62	875		46	891		13	924		28	909	

Others: Cough, anorexia, backache, abdominal pain; * *p* < 0.05. (Note: cases with dual positivity for SFG and TG have been included in both SFG and TG.)

**Table 4:** Association of different signs and symptoms against rickettsioses and other causes of undifferentiated fever.

Signs and Symptoms		Diagnosis		*p* Value
		Rickettsioses	Non-Rickettsioses	
Rash	Yes	43	220	0.559
	No	116	665	
Eschar	Yes	31	9	<0.001 *
	No	128	876	
Headache	Yes	129	676	0.190
	No	30	209	
Arthralgia	Yes	106	517	0.051
	No	53	368	
Myalgia	Yes	53	245	0.149
	No	106	639	
Lymphadenopathy	Yes	8	25	0.145
	No	151	858	
Others	Yes	19	140	0.443
	No	140	797	

Others: Cough, anorexia, backache, abdominal pain; * *p* < 0.05.

**Table 5:** Association of age, gender, occupation, and district of residence with acute STG cases.

Variables	OR	95% CI		*p* Value
Age group (yrs.)				
Children	Ref.			
13–24	1.00	0.43	2.32	0.995
25–36	0.80	0.34	1.87	0.608
37–48	1.81	0.82	3.99	0.140
Above 48	1.08	0.46	2.54	0.856
Gender				
Male	Ref.			
Female	0.88	0.54	1.42	0.596
Occupation				
Farmer	Ref.			
Office worker	1.08	0.58	2.00	0.802
Student	0.43	0.20	0.93	0.031 *
Housewife	0.85	0.39	1.86	0.683
Unemployed	0.79	0.20	6.26	0.821
Pre-school	1.31	0.47	3.66	0.604
District				
Samtse	Ref.			
Chukha	0.92	0.37	2.30	0.862
Thimphu	1.45	0.59	3.59	0.416
Dagana	1.08	0.37	3.16	0.887
Tsirang	0.61	0.13	2.91	0.532
Sarpang	1.19	0.49	2.93	0.699
Zhemgang	1.36	0.43	4.27	0.597
Mongar	0.52	0.14	1.98	0.337
Pemagatshel	0.94	0.28	3.17	0.915
Trashigang	0.47	0.10	2.24	0.341
Samdrupjongkhar	0.57	0.17	1.92	0.367

OR: odds ratio; 95% CI: 95% confidence interval; * *p* < 0.05.

## Supplementary Materials

The following are available online at http://www.mdpi.com/2414-6366/3/1/12/s1, Supplementary S1: Raw data, Supplementary S2: Table showing monthly cases of SFG, TG, STG and QF with corresponding average month temperature and rainfall of Bhutan.
