# Supplementary material for: Rickettsial Infections and Q Fever Amongst Febrile Patients in Bhutan
Source: Trop Med Infect Dis. 2018 Jan 25;3(1):12. doi: 10.3390/tropicalmed3010012 (PMC6136613; doi:10.3390/tropicalmed3010012)
Supplement: Supplementary file 1 [file tropicalmed-03-00012-s001.zip › tropicalmed-260273-supplementary/supple 2.pdf]

Supplementary table 1: Monthly cases of SFG, TG, STG and QF with corresponding average month temperature and rainfall of Bhutan

Months	SFG	TG	STG	QF	Temperature (C)	Rainfall (mm)
Oct-14	13	2	30	3	18	53
Nov-14	5	3	11	6	13	10
Dec-14	4	0	4	3	10	4
Jan-15	4	2	2	3	8	11
Feb-15	3	2	5	2	10	26
Mar-15	5	0	4	3	13	26
Apr-15	5	1	3	3	16	56
May-15	8	1	6	2	19	103
Jun-15	9	3	5	4	21	182

SFG: Spotted fever group; TG: Typhus group; STG: Scrub typhus group; QF: Q fever; C: degree Celsius; mm: millimeter
